# Flexible Liquid Crystal Polymer Technologies from Microwave to Terahertz Frequencies

**DOI:** 10.3390/molecules27041336

**Published:** 2022-02-16

**Authors:** Zepeng Zhou, Wenqing Li, Jun Qian, Weihong Liu, Yiming Wang, Xijian Zhang, Qinglei Guo, Yevhen Yashchyshyn, Qingpu Wang, Yanpeng Shi, Yifei Zhang

**Affiliations:** 1 Shandong Technology Center of Nanodevices and Integration, School of Microelectronics, Shandong University, Jinan 250100, China; zhouzepeng1997@gmail.com (Z.Z.); 14718246663@163.com (W.L.); jun.qian@mail.sdu.edu.cn (J.Q.); wym@sdu.edu.cn (Y.W.); zhangxijian@sdu.edu.cn (X.Z.); qlguo@sdu.edu.cn (Q.G.); wangqingpu@sdu.edu.cn (Q.W.); 2 School of Electronic Engineering, Xi’an University of Posts & Telecommunications, Xi’an 710121, China; liuweihong1980@163.com; 3 Institute of Radioelectronics and Multimedia Technology, Warsaw University of Technology, 00-665 Warsaw, Poland; Y.Yashchyshyn@ire.pw.edu.pl

**Keywords:** liquid crystal polymer, multilayer circuit, antenna, package, flexible, MEMS, biomedical, microfluidics

## Abstract

With the emergence of fifth-generation (5G) cellular networks, millimeter-wave (mmW) and terahertz (THz) frequencies have attracted ever-growing interest for advanced wireless applications. The traditional printed circuit board materials have become uncompetitive at such high frequencies due to their high dielectric loss and large water absorption rates. As a promising high-frequency alternative, liquid crystal polymers (LCPs) have been widely investigated for use in circuit devices, chip integration, and module packaging over the last decade due to their low loss tangent up to 1.8 THz and good hermeticity. The previous review articles have summarized the chemical properties of LCP films, flexible LCP antennas, and LCP-based antenna-in-package and system-in-package technologies for 5G applications, although these articles did not discuss synthetic LCP technologies. In addition to wireless applications, the attractive mechanical, chemical, and thermal properties of LCP films enable interesting applications in micro-electro-mechanical systems (MEMS), biomedical electronics, and microfluidics, which have not been summarized to date. Here, a comprehensive review of flexible LCP technologies covering electric circuits, antennas, integration and packaging technologies, front-end modules, MEMS, biomedical devices, and microfluidics from microwave to THz frequencies is presented for the first time, which gives a broad introduction for those outside or just entering the field and provides perspective and breadth for those who are well established in the field.

## 1. Introduction

A liquid crystal is a kind of intermediate-state material combining the fluidity of a liquid with the ordering of a crystal [1,2,3]. Liquid crystal polymers (LCPs) are polymers that display the properties of liquid crystals under certain conditions. According to the conditions of liquid crystal formation, LCPs are typically classified into two categories, i.e., thermotropic LCPs and lyotropic LCPs. The former form the liquid crystalline state above the glass transition temperature or at the melt temperature, while the latter achieve the same state in solution [2]. Currently, most of the thermotropic LCPs are composed of aromatic polyesters with polycondensation. The typical monomer structures for LCPs include monomers with different chain lengths, monomers with substituents, rigid bending monomers, crankshaft monomers, and rotational monomers, which comprise small molecules after polycondensation [2]. The synthesis of aromatic polyesters can be achieved with four basic synthesis reactions, i.e., Schotten–Bauman reaction, high-temperature melt transesterification, oxidative esterification, and phenyl ester reaction, the pros and cons of which have been discussed in detail in [4]. In recent years, thermotropic LCPs have gained great attention as promising microwave, millimeter-wave (mmW), and terahertz (THz) circuit substrates and packaging materials for fifth-generation (5G) and Internet of Things (IoTs) applications [5,6,7]. For convenience, we use LCPs to represent thermotropic LCPs in the rest of this paper. Early in the 1990s, LCPs were considered as possible candidates for microwave applications [8]. However, the tearing problem during manufacturing process makes the film uniformity unsuitable for circuit fabrication. To overcome this problem, a biaxial die extrusion (BDU) method was developed to apply uniform strength [8,9]. Additionally, the LCP substrates were difficult to process due to the bad metal adhesion and poor reliability of the plated vias in the early stage. The adhesion problem was solved by using an optimized extrusion angle and rate in the developed BDU process, making the coefficient of thermal expansion (CTE) of LCP films compatible with several commonly used metals and semiconductors, such as copper, gold, and titanium [10]. On the other hand, many studies have focused on optimizing surface treatments as well as hole drilling and de-smearing methods [11,12]. These process obstacles of LCP films were not fully overcome until 2002 [10]. Since then, LCP films with copper claddings have become commercially available [13], and the use of flexible LCP technologies has been rapidly increasing.

The early studies mainly investigated the material properties of LCP films during the beginning of the twenty first century [10,14], such as the dispersive dielectric constant *ε_r_* and loss tangent *tanδ*, water absorption, package hermeticity, thermal stability, and bending effect. Sooner after, hundreds of functional passive devices, such as filters and antennas, were developed in single-layer and multilayer LCP films, which typically do not require extra bias voltage to work [7,15]. Meanwhile, the integration and packaging of passive and active chips with LCP passive circuits became of great interest, which enabled the commercial application of LCP devices in advanced wireless systems at microwave and mmW frequencies [5,6]. In 2019, the application of flexible LCP antennas and circuits in the Apple iPhone made LCPs well known to the public, which inspired many review articles on LCP technologies relating to various aspects over last two years [5,6,7,15]. Gu et al. reviewed antenna integration and packaging technologies on LCP substrates in 2020 [5], while Watanabe et al. discussed LCP technologies in front-end package applications in 2021 [6], both of which could be used in fifth-generation (5G) wireless communication. Ji and colleagues summarized the polymerization, chemical structure, aggregated state, modification, and processing of typical LCPs relating to aspects of materials science in 2020 [15], while Khan et al. surveyed the mechanical properties of wearable antennas on LCP and other flexible films in 2021 [7]. In addition to wireless applications, LCP technologies have been developed for many other promising applications, such as micro-electro-mechanical systems (MEMS) [16], biomedical electronics [17], and microfluidics [18]. However, to the best of the authors’ knowledge, no articles have reviewed the LCP technologies thoroughly to date.

**Figure 1:**
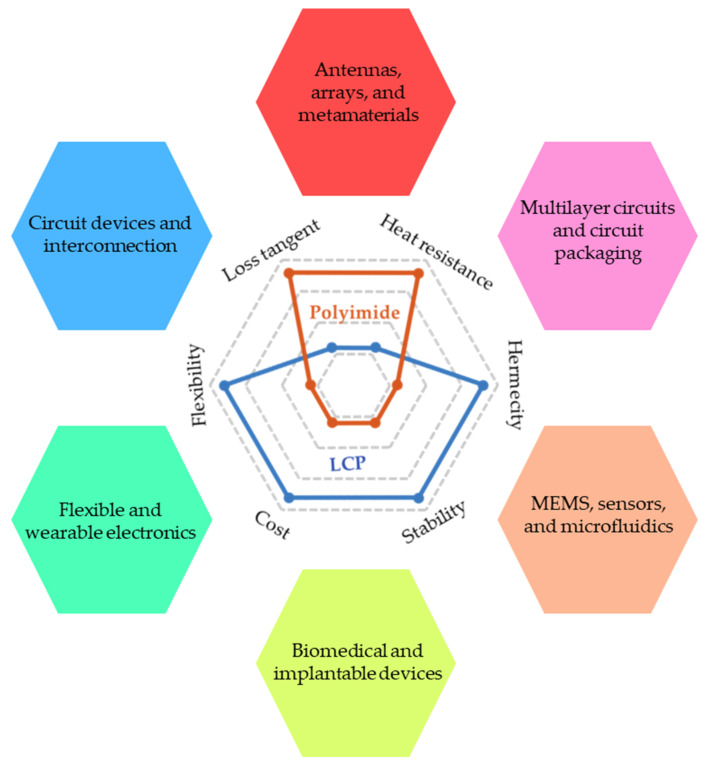
Material properties and promising applications of LCP technologies.

In this paper, we provide a comprehensive review of LCP technologies, covering the basic material properties; flexible circuit components and antennas; integration and packaging technologies; front-end modules; and cutting-edge wireless applications at microwave, mmW, and THz frequencies, as well as other emerging applications. These promising applications are mainly attributed to the outstanding material properties of LCP films, as shown in Figure 1. The small dielectric constant and low loss tangent enable electromagnetic devices with low loss and high efficiency rates, such as antennas, transmission lines, and filters, ranging from several MHz up to several hundred GHz. The multilayer lamination and good hermeticity make LCPs promising packaging materials for microwave and millimeter-wave monolithic integrated chips (MMICs), MEMS, implantable devices, and microfluidics. Furthermore, their flexibility and small thickness are advantageous in conformal and wearable applications, as well as flexible interconnections. The important companies that incorporate LCP technologies into materials, fabrication technologies, and electronic products are listed in Table 1. Lastly, the mechanical stiffness, chemical resistance, and biocompatibility make LCP devices suitable for long-term implantable applications. We expect this review to give an interesting introduction for those new to the field and to show breadth, depth, and perspective for researchers in this field.

## 2. Material Properties and Device Fabrication

### 2.1. Material Properties

#### 2.1.1. Electromagnetic Properties

As non-magnetic insulator, printed circuit board (PCB) substrates typically have two important electromagnetic parameters, i.e., a dielectric constant *ε_r_* and loss tangent *tanδ*. At low microwave frequencies, such as several GHz, the most popular printed circuit board (PCB) materials are FR4 and polyimide, which typically have a high loss tangent range of >0.025 at 10 GHz [19]. Their loss tangent further increases as the frequency increases. The water absorption rates of FR-4 and polyimide are as high as 0.15% and 1–5%, respectively [19]. Both the high loss tangent and the high water absorption impede the high-frequency and packaging applications of FR-4 and polyimide. As a high-frequency alternative to traditional PCB materials, LCPs have a low loss tangent (tanδ) range of 0.002–0.005 below 110 GHz and a low water absorption rate of <0.04% [10,20]. The loss tangent remains low at THz frequencies, i.e., 0.0055–0.009 from 110 to 170 GHz [21], 0.009–0.012 from 170 to 330 GHz [22], and less than 0.09 up to 1.8 THz [23]. On the other hand, the dielectric constant range of LCP films is 3–3.5 from DC up to 1.8 THz [10,11,21,22,23], which is suitable for antenna applications to improve the radiation efficiency.

Many technologies have been developed to characterize the dielectric constant and loss tangent of LCP films. Zou et al. employed a transmission-line-based resonator method in 2002, which provides accurate data at discrete spectral points, i.e., the resonant frequencies [14]. Thompson et al. measured the scattering parameters of cavity resonators and transmission lines for extracting both discrete and continuous electromagnetic parameters in 2004, which provides a good reference for device design up to 110 GHz [10]. To obtain precise data, the radiation loss should be considered in the calculation. At THz frequencies, i.e., above 100 GHz, a transmission line resonator method was developed for D-band characterization, and Monte Carlo uncertainty analysis was combined to obtain better accuracy [21]. In 2015, Gao et al. used microstrip lines on 50-μm-thick LCP substrates to characterize the dielectric constant and loss tangent from 220 to 330 GHz [22]. These properties have been characterized up to 1.8 THz by using THz spectroscopy [23]. High-accuracy methods for characterizing LCP properties are still being investigated [24,25].

#### 2.1.2. Mechanical Properties

As flexible films, LCPs have found many important applications in conformal and wearable antennas, as reviewed in [7]. Furthermore, LCP films are suitable for many other devices. Typically, the mechanical strength and elastic modulus of LCP are comparable to other common plastics [7]. Its mechanical stability is 4 times higher than that of polyimide, meaning that the misregistration and layer-to-layer misalignment are much lower during the fabrication process [14], which is important for mmW and THz devices requiring high fabrication resolution. Additionally, LCPs exhibit relatively higher stiffness than polyimides and parylenes, which enables the application of flexible neurol probes and stimulators [17].

#### 2.1.3. Other Properties

LCPs have high chemical and flame resistance [14], good dielectric stability at high temperatures up to 100 °C [26], a near-hermetic nature [20], and low cost [10]. Due to the reel-to-reel manufacturing, the cost of LCPs is as low as that of traditional FR4. Another attractive factor is that the two types of LCP materials have different melting temperatures, i.e., 315 and 290 °C [10]. In this case, LCP films were laminated onto multilayer circuits, whereby the high-temperature films were used as core layers and the low-temperature films were used as a bond ply layer. Alternatively, low-temperature LCP layers may be laminated directly at around 290 °C, such as Panasonic FELIOS LCPs, which slightly varies the film thickness.

### 2.2. Fabrication of LCP Devices

The commercially available LCP films are laminated with copper claddings, which are suitable for standard photolithography and embossing with lasers or drills [27]. Typically, liquid photoresist techniques are preferred for small samples and dry-film techniques are used for large panels, both of which should be followed by the wet-etching process to pattern metals. These standard PCB fabrication technologies are available in many PCB manufacturers, such as Fujikura in Japan and AKM Electronics Industrial (PanYu) Ltd. in China (see Table 1). To obtain low resolutions for metal patterns, lift-off techniques with titanium or palladium seed layers may be utilized [12,27].

Recently, inkjet printing with silver nanoparticles has gained great interest for fabricating LCP devices, which is a low-cost, rapid, and simple approach [28,29,30,31]. A coupled line filter at 25 GHz and dipole antenna at Ka-band were printed with silver particles in 2013 and 2014, respectively [29,30]; however, the conductor loss is non-negligible as the frequency increases. To minimize the conductor loss, highly conductive silver layers and lithography–inkjet printing hybrid methods have been investigated [32,33,34], showing reasonable loss up to 110 GHz for the transmission lines. Another big concern with inkjet printing is the degradation of conductor loss with the bending effect, which has been studied in [31].

## 3. Flexible LCP Circuits

**Figure 2:**
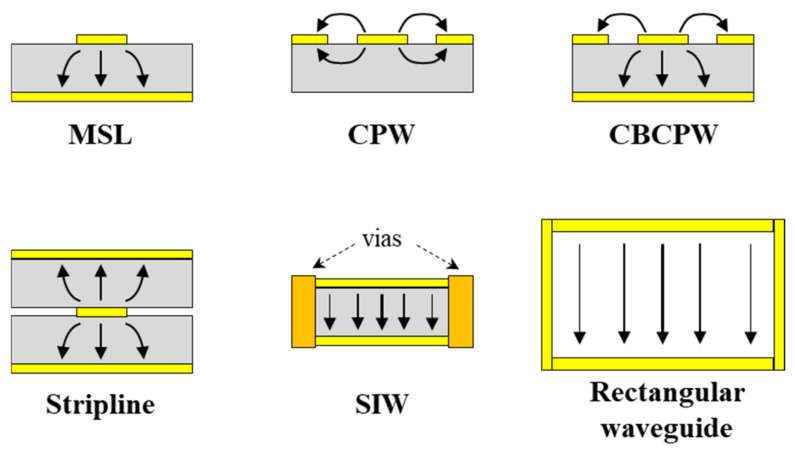
Fundamental mode profiles of transmission lines and waveguides.

One of the most important components in a circuit is the transmission line, whose propagation loss and reflection loss may dominate the power consumption in mmW and THz modules as the frequency increases. Most traditional transmission lines are fabricated on LCP films, such as microstrip lines (MSLs), co-planar waveguides (CPWs), striplines (SLs), conductor-backed CPWs, and substrate-integrated waveguides (SIWs) [19], whose fundamental mode profiles are illustrated in Figure 2. In multilayer circuits, MSLs and conductor-backed CPWs are suitable for use in the surface layers due to their shielding ground, and SLs and SIWs are more appropriate in the inner layers due to their perfect layer-to-layer electrical shielding [35,36,37]. CPWs may be desired for the integration of MMICs, whose mode profile is the same as the ground–signal–ground (G-S-G) pads of MMICs. Recently, a novel substrate-integrated coaxial line (SICL) was reported for antenna array feeding on multilayer LCP substrates [38]. Based on various transmission lines, functional circuit devices, such as filters, duplexers, baluns, and power dividers, have been proposed, and their flexible applications have been investigated.

### 3.1. Transitions

**Figure 3:**
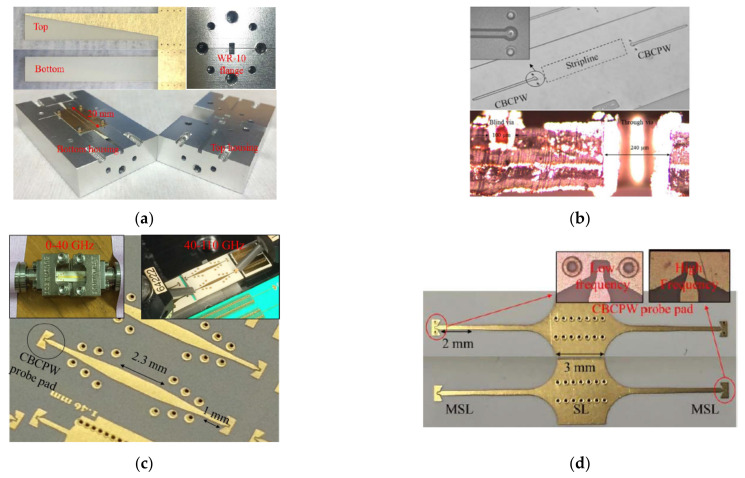
Multilayer LCP circuit interconnections. (**a**) WG-to-SIW transition with linearly tapered antipodal slot line in multilayer LCP substrates in the W-band [42]. Copyright IEEE, 2017. (**b**) Vertical CBCPW-to-SL transition with blind and through vias [43]. Copyright John Wiley and Sons, 2015. (**c**) Ultra-wideband MSL-to-MSL transitions in different circuit layers in a multilayer LCP substrate [44]. Copyright IEEE, 2017. (**d**) Vialess MSL-to-SL transition in a multilayer LCP substrate in E- and W-bands [45]. Copyright IEEE, 2017.

A transition is a passive transformation structure that connects circuit components with different topologies, which is of great importance for connecting the transmission lines and output interfaces with various mode profiles and impedances, as shown in Figure 2. The air-filled rectangular waveguide (WG) is one of the most important structures for interconnecting and interfacing in microwave and mmW systems due to its low loss and high power-handling capability [37]. The transitions from planar LCP circuits to rectangular WGs have been investigated widely. Ariffin and Isa reported a broadband WG-to-MSL transition on an LCP substrate with an optimized ground plane and a triple-patch probe in the E-band [39], while Zhang et al. proposed a slot-coupled WG-to-MSL transition with a resonant patch in the W-band [40]. As a low-profile alternative to a rectangular WG, an SIW is a WG-like structure fabricated in a dielectric substrate by using two rows of metal vias to connect the top and bottom conductor layers [37]. Low-loss transitions from MSL, CPW, and CBCPW to SIW transitions were developed at mmW frequencies in previous studies [19,41]. In [42], a broadband WG-to-SIW transition in multilayer LCP substrates was reported in the W-band, as illustrated in Figure 3a, which utilized a linearly flared antipodal slot line to suppress the high-order modes and guide signals to the right circuit layer.

In multilayer circuits, the transmission lines in different layers are typically linked using vias or aperture coupling. An ultra-wideband (UWB) CBCPW-to-SL transition with vias was reported from 0 to 100 GHz [43], as shown in Figure 3b. However, the large annual ring for the surface finish of the vias induces significant reflection and insertion loss at the W-band. To avoid using vias, the use of UWB MSL-to-MSL and MSL-to-SL transitions with smooth mode-conversion structures was reported in the W-band [44,45], as illustrated in Figure 3c,d, respectively, achieving low insertion loss rates of less than 1.2 dB up to 110 GHz. A conductor-backed CPW-to-SICL vertical transition was developed for array-feeding networks in 2017 [38]. The SICLs allow the easy design of antenna array feeding networks as MSLs and good electromagnetic compatibility in SIWs. In addition, it is interesting to note that inkjet printing, i.e., an additive manufacturing technology, was developed for 3-D transitions, achieving a minimal feature size of as small as 20 μm and a UWB bandwidth of 0.1 to 40 GHz [46].

### 3.2. Filters

**Figure 4:**
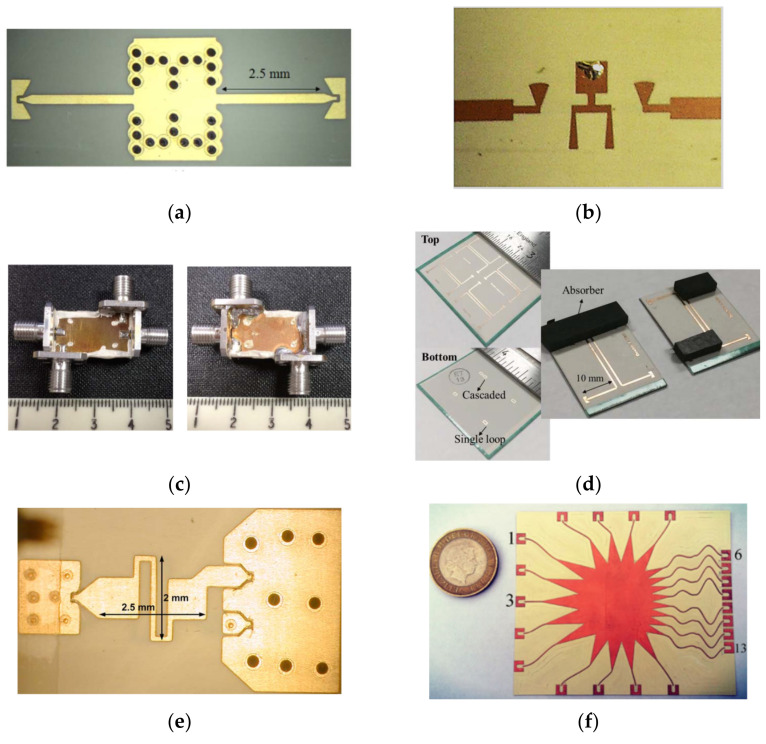
Filters and other circuit devices on LCP films. (**a**) SIW filter with iris windows in a single-layer LCP film at 94 GHz [50]. Copyright John Wiley and Sons, 2016. (**b**) Compact eight-pole bandpass filter with transmission zero from 2 to 10 GHz in multilayer LCP substrates [52]. Copyright IEEE, 2008. (**c**) Miniaturized balanced bandpass filter with full EM shielding using self-package technologies in multilayer LCP substrates [62]. Copyright IEEE, 2019. (**d**) Slot-coupled traveling-wave directional filters with four ports at 95 GHz in multilayer LCP substrates [63]. Copyright IEEE, 2017. (**e**) Compact broadband balun based on a quasi LC transformer network [65]. Copyright John Wiley and Sons, 2013. (**f**) Low-profile Rotman-lens-fed beamformer for 5G conformal applications at 28 GHz [66]. Copyright John Wiley and Sons, 2019.

**Figure 5:**
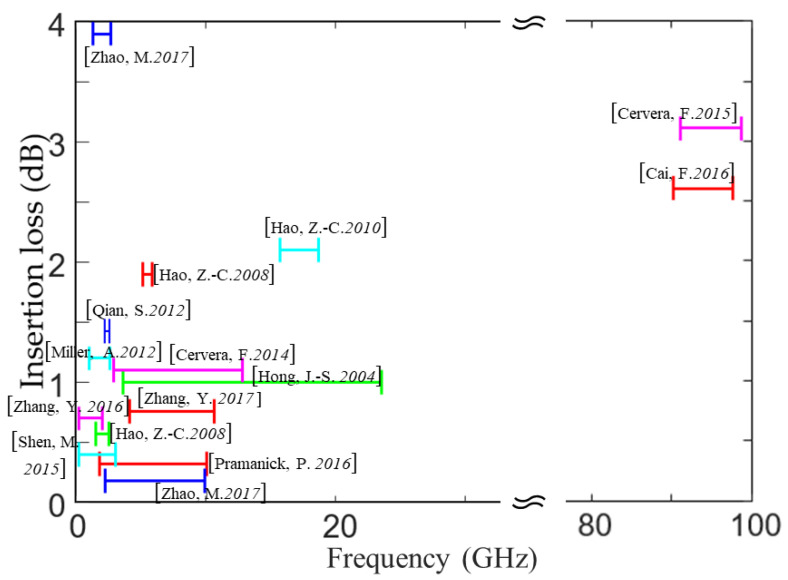
Loss and bandwidth properties of LCP filters [45,46,47,48,49,50,51,52,53,54,55,56,57,58,59].

Filters reject the unwanted signal frequencies and permit good transmission of the desired frequencies, which can be found in virtually any type of wireless communication, radar, radiometry, or measurement system at microwave or millimeter-wave frequencies [19]. According to their spectral responses, filters typically can be classified into four different categories, i.e., low-pass, high-pass, band-pass, and band-stop [47]. The design principles and mechanisms for various filters have been extensively studied to date [48]. Once commercial LCP films became available, planar filters based on MSLs and SIWs were investigated in single-layer LCP substrates [49,50], with SIWs illustrated in Figure 4a. To achieve compact size, various vertical structures were designed for high-pass, low-pass, and band-pass filters in multilayer LCP substrates [51,52,53,54,55,56] (see Figure 4b). Recently, self-packaging techniques were developed for 3-D filter fabrication by Hong’s group at Heriot-Watt University, which are typically suitable for applications at low microwave frequencies, e.g., less than 15 GHz [57,58,59,60,61,62], as shown in Figure 4c. In addition to the two-port filters with either a pass band or stop band, a directional filter with four ports was developed with little reflection in a multilayer LCP substrate at 95 GHz, as shown in Figure 4d, achieving a reflection loss of more than 10 dB in the W-band [63]. These directional filters can be used for suppressing oscillation in front-end modules and may be cascaded in series for frequency division multiplexing [64]. The statistic insertion loss and bandwidth for various filters on LCP films are depicted in Figure 5. It can be seen that most of the reported filters were developed below 20 GHz. We expect more high-frequency filters to be developed for 5G applications and beyond in the next few years.

### 3.3. Other Circuit Components

In addition to the aforementioned circuit transitions and filters, many other functional devices have been developed in LCP films. Ta et al. reported a compact broadband balun in 2013 [65] (see Figure 4e) and Pham et al. reported a UWB low-loss balun with stacked-defected grounding in 2018 [67], both of which are preferable for antenna design. A long-time-delay circuit with a broad bandwidth was reported in [68], whose 2-bit relative delay reached up to 600 ps. Chieh and Pham developed a flexible Wilkinson power divider with integrated thin-film resistors, achieving a wide bandwidth ratio of 9:1 within a compact size [69]. In 2018, Rahimian et al. proposed the first experimental mmW beamformer based on the Rotman lens, achieving a low profile, wide band, and good flexibility [66], as shown in Figure 4f.

## 4. Flexible Antennas and Arrays

An antenna connects the electromagnetic waves propagating in free space and the guided waves in transmission lines and waveguides, which are key elements for wireless functions [70]. An antenna array is a collection of antennas used to achieve versatile functions, such as gain enhancement, beam steering, and multi-input–multi-output (MIMO) functionality [70]. Another way to manipulate electromagnetic waves in free space is by using metamaterials (MTMs), which are artificial structures with unnatural optical properties, such as negative permittivity and permeability [71]. Combining antennas and MTMs may result in many fantastic properties, such as miniaturization, gain enhancement, and high electromagnetic compatibility (EMC). LCPs are perfect materials for antenna and metamaterial applications, due to their low dielectric constant, small loss tangent, flexibility, light weight, and low cost [5,7]. In the following of this section, we will review various antennas, metamaterials, and arrays on LCP substrates.

### 4.1. Antennas

**Figure 6:**
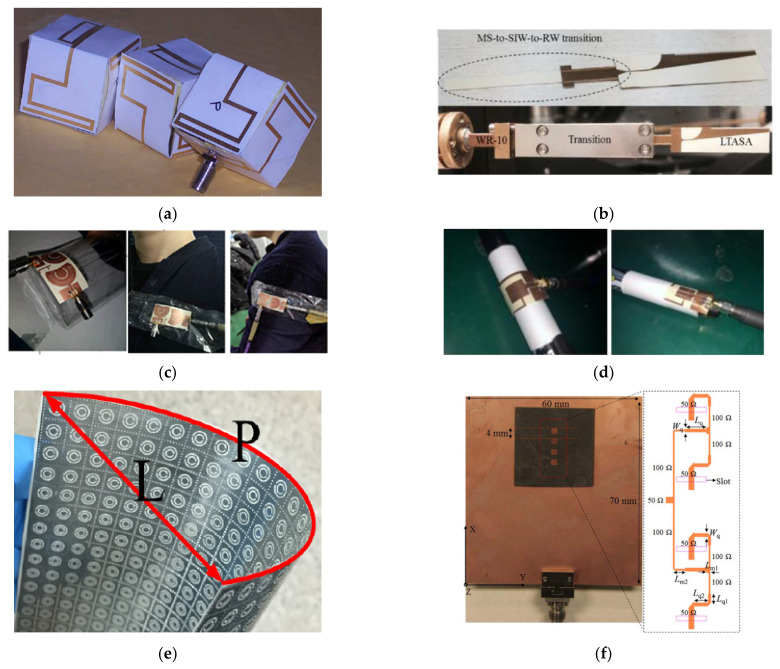
Flexible LCP antennas and MTMs. (**a**) Miniaturized 3-D cubic antenna with folded meander line and truly omnidirectional radiation patterns at 915 MHz [73]. Copyright IEEE, 2009. (**b**) TSA from 47 to 110GHz on a thin-film LCP characterized with an optical far-field measurement system [77]. Copyright IEEE, 2016. (**c**) CPW-fed dual-band-notched MIMO flexible antenna and its bending test [83]. Copyright John Wiley and Sons, 2021. (**d**) Compact tri-band antenna with L-shaped strips on the main radiation patch and the ground plane and its bending test [85]. (**e**) Flexible microwave polarizer based on complementary split ring resonators at 10 GHz [87]. (**f**) Phased patch array antenna with continuous beam steering angles from 31 to 39 GHz [88]. Copyright John Wiley and Sons, 2016.

Investigation of flexible LCP antennas began in 2005, i.e., soon after commercial LCP films with copper lamination became available [72]. Since then, various antennas have been developed for flexible and wearable applications. In 2009, Kruesi reported a folded 3-D cubic antenna in the ultra-high frequency (UHF) range for radio frequency identification devices (RFIDs) and wireless sensor networks (WSNs) [73] (see Figure 6a). In 2010, an aperture-coupled staked patch antenna with millimeter-thick micromachined photoresist spacers was developed, achieving a high radiation efficiency of 97% at 10 GHz [74]. For high-gain applications, flexible tapered slot antennas (TSAs) have been developed on LCP substrates [75,76]. In 2016, Zhang et al. demonstrated the potential of LCP TSAs for THz applications [77], as shown in Figure 6b. With the emergence of 5G technologies, multi-band and UWB omni-directional antennas with good flexibility have gained great interest in recent years [78,79,80,81,82,83,84,85,86]. In 2018, Madhav et al. developed a compact MIMO antenna for automotive communication, achieving low mutual coupling between antenna elements [82]. Monopole antennas designed with notch structures were used with ultrawide bandwidths for wireless local area network (WLAN), MIMO, and Worldwide Interoperability for Microwave Access (WiMax) applications in 2021 [83], as shown in Figure 6c. Furthermore, compact multiband antennas have also been investigated for flexible communication and implantable applications in the last two years [84,85,86].

**Figure 7:**
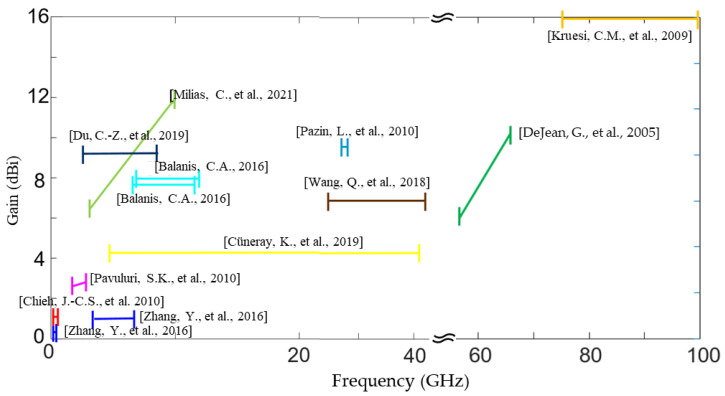
Gain and bandwidth properties of LCP antennas [69,70,71,72,73,74,76,77,78,79,80].

Since the early 2000s, the bending effect has been investigated for flexible LCP antenna applications. The bending degree can be described via bending angles or radius. A double-exponential TSA was folded with an angle of 18° in the H-plane in 2006, showing nearly identical patterns in the E-plane and an off-axis beam in the H-plane, as well as enlarged cross-polarization [75]. Recently, low-gain omni-directional antennas were also investigated for bending applications. Typically, extensive bending with a small curvature radius sacrifices the reflection loss [84,85]. However, the degradation can be suppressed via careful design, such as the use of L-shaped strips on both the main radiation patch and the ground plane (Figure 6d), as reported in [86]. The discrepancies between simulation and measurement for flexible antennas are discussed in [89], and the bending analysis is reviewed in [7]. In this case, we are not going to discuss on the bending effect. Interested readers may refer to previous studies [7,89]. The performance data for LCP antennas are illustrated in Figure 7.

### 4.2. Metamaterials

Metamaterials are artificial subwavelength structure arrays, which can manipulate the propagation of electromagnetic waves, such as the amplitude, frequency, phase, and polarization [90]. They allow a strong light–matter interaction enhancement and an abnormal refractive index that does not exist in natural materials [90,91,92,93], which enables many unprecedented phenomena, including cloaking, a flat lens, and perfect absorption. Flexible MTMs are of considerable importance for conformal systems [93]. LCPs are promising candidates for flexible MTMs due to their flexibility, small thickness, low dielectric constant, and low loss tangent at mmW and THz frequencies. In 2010, a metallic square array was proposed to achieve a wide range of variation in its effective dielectric constant in the W-band [94]. In 2021, Li et al. reported on the use of frequency-selective surface (FSS) and complementary split-ring resonators (SRRs) on an LCP film in the X-band [87], as shown in Figure 6e. The curved LCP increases the light transmission loss from 0.75 to 3 dB with a bending radius of 8.8 cm. However, the resonant frequency is not sensitive to the bending.

Additionally, MTMs have been widely applied in LCP antennas [71]. SRRs have been designed on the antenna feeding lines to broaden bandwidth [95], and complementary SRRs have been used to obtain notch-band characteristics for UWB LCP antennas on the ground electrode [96,97]. In 2014, Soliman et al. reported on the use of electromagnetic band gap (EBG) structures to improve the bandwidth, reduce the size, and improve the gain of a planar inverted F antenna (PIFA) [98].

### 4.3. Antenna Arrays

An antenna array is a set of antennas working together as a single antenna, whose radiation beams are the superposition of individual antenna elements. Antenna arrays can achieve high gain and directivity, allowing beam forming, beam steering, and sidelobe and backlobe suppression [70]. Typically, the array element is a low-gain antenna with a large beam width, such as a patch antenna, slot antenna, or dipole antenna. Early in 2008, Kingsley et al. reported on a phased patch array with a MEMS phase shifter on a multilayer LCP substrate at 14 GHz, achieving a beam steering angle of 12° [99]. To improve the bandwidth of the patch antenna, a stacked patch array was developed for mmW applications [88,100]. In 2013, Samuel Chieh et al. reported on a stacked patch with a substrate-embedded air cavity for isolation improvement, whose bandwidth ranges from 74 to 97 GHz, i.e., 27% centered at 85 GHz [100]. In 2016, Zhang et al. reported on a phased patch array with a bandwidth of 23% and continuous beam steering up to 33° from 31 to 39 GHz [88], as shown in Figure 7f. In 2017, Xing et al. used SICLs for a patch array to suppress backlobes and sidelobes [38]. Additionally, slot arrays with various coupling elements have been proposed for beam forming in WLAN applications [101]. We note that an MTM coupling element was reported for an antenna design in [102]. Dipole arrays with considerable bandwidth have gained interest for 5G applications recently [103]. Gu et al. reported on a magnetoelectric dipole array with an antenna-in-package (AiP) design for a phased-array module in 2021 [104]. With the development of 5G technologies, studies on flexible arrays and bending effects have been conducted within the last two years [7].

## 5. Integration and Packaging Technologies

The atmospheric attenuation of electromagnetic waves enlarges as the frequency increases. To achieve high detection sensitivity and cover a large dynamic range, high-gain and low-noise figure modules are of great interest for use in wireless systems at mmW and THz frequencies, such as in 5G communication, imagers, and radars [19]. These advanced systems typically comprise multiple MMICs and numerous passive lumped components with sophisticated integration and packaging technologies. Recently, system-in-package (SiP) and system-on-package (SoP) techniques have received significant attention in the regime of antenna-in-package (AiP) and front-end modules [5,6,7,8]. The use of SiP can allow high density, low profile, low loss, and light weight with a multilayer carry board [5,104]. LCP and LTCC SiP technologies were compared in [105]. The development of 5G technologies, high-frequency and multi-chip module (MCM) integration, and packaging is becoming increasingly important in this field.

### 5.1. Chip Integration and Packging

The study of chip integration on flexible LCP substrates began with passive chips. Zhou et al. first investigated the integration of a gallium arsenide (GaAs) MMIC switch with MSL in 2002, and then reported the integration of capacitors and resistors on an LCP substrate in 2005 [14,106]. Mukherjee et al. studied inductors and bandpass filters embedded in multilayer LCP substrates in 2005 [107], while Bavisi et al. reported on LC oscillators embedded in multilayer LCP substrates in 2006 [108]. High-Q-embedded passives on large-panel multilayer LCP substrates were investigated in 2007 [109], which eliminate the effects of pads and through-hole interconnection with the de-embedding method. Recently, inkjet-printed inductors and capacitors have been reported as alternatives to the traditional bulky surface-mounted ones [110].

On the other hand, active MMICs integrated in flexible LCP substrates were investigated a little later than the passive chips. In 2006, Thompson et al. studied integrated GaAs in a low-noise amplifier (LNA) in multilayer LCP substrates and near-hermetic packaging in the mmW range [111]. In 2008, the surface-mounted integration and packaging of LNA in the Ka-band was reported, demonstrating a measured leak rate of as low as 3.6 × 10^−8^ atm·cc/s [112]. Conformal inverse L-shaped monopole antenna integrated and packaged for WLAN/WiMax applications was reported at 5 GHz in 2009 [113], introducing a method for the direct integration of a large antenna element into a small module package. In 2013, Chlieh et al. integrated gallium nitride (GaN) amplifiers and microfluidic cooling in a multilayer LCP substrate for high-power applications at 5 GHz [114]. In 2017, Zhang et al. developed an MCM integration and packaging approach in the W-band, achieving a high gain of 50 dB, a low noise figure of less than 6 dB, and a linear phase from 80 to 97 GHz [115]. Jiang et al. reported on the use of LCP packages for miniaturized magnetoelastic resonators for tagging applications in 2019, expanding the application ranges of LCPs [116].

### 5.2. Integraion Technologies

**Figure 8:**
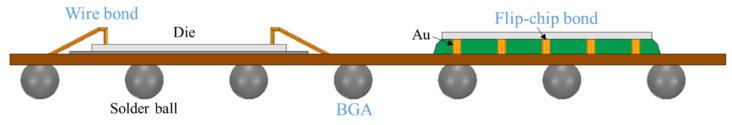
Configuration of BGA, wire bonding, and flip-chip bonding techniques.

**Figure 9:**
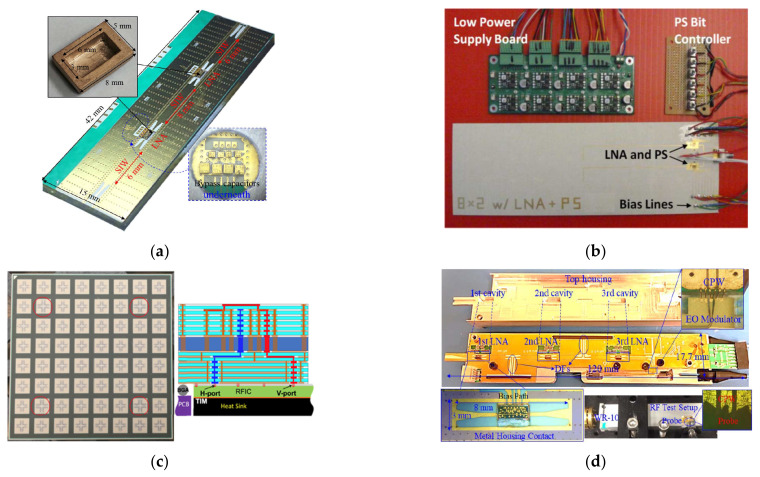
System-in-package and antenna-in-package modules. (**a**) Packaged double low-noise amplifier module with V-shaped wire bond in 3-D-printed housing in the W-band [115]. Copyright IEEE, 2017. (**b**) Packaged phased array with integrated silicon germanium amplifiers and phase shifters in the X-band [121]. Copyright IEEE, 2011. (**c**) AiP phased-array module with wide bandwidth for 5G applications [104]. Copyright IEEE, 2021. (**d**) Front-end multi-chip receiving module for passive mmW imaging, achieving a high gain rate of 63.5 dB and a low noise rate of less than 6 dB at 95 GHz [129]. Copyright IEEE, 2018.

Typically, ball grid array (BGA), wire bonding, and flip-chip bonding techniques are suitable for chip integration at microwave and mmW frequencies, as illustrated in Figure 8. The BGA and flip-chip bonding are vertical connections, while the wire bonding is a planar connection. Yazdani studied BGA packaging in 2006 [117], showing increasing insertion loss as the frequency increases due to the considerable conductor loss of solder balls. The insertion loss was as large as 10 dB at 40 GHz. As an alternative, wire bonding shows better high-frequency performance, whose equivalent circuit model is well discussed in [118]. With a low bonding profile, i.e., small wire length and lift height, wire bonding can allow broadband low-loss properties up to 77 GHz [119] and narrowband low-loss properties up to 122 GHz [120]. Zhang developed V-shaped wire bonding techniques for the integration of wideband indium phosphide (InP) LNA on LCP substrates in 2017, achieving low insertion loss up to 110 GHz [115], as shown in Figure 9a. Due to the small interconnect profile, flip-chip bonding addresses the low loss by using gold balls or posts at mmW and THz frequencies [121,122,123,124]. Khan et al. investigated flip-chip interconnections with capacitance compensation from DC ranges up to 170 GHz [122]. However, the parasitic modes need to be considered in the integration design.

### 5.3. Front-End Modules

With the development of integration and packaging techniques, various front-end modules with LCP carrier boards have been developed for advanced wireless applications. Patterson et al. developed phased arrays with amplifiers and phase shifters for X-band detection in 2011 [125], as shown in Figure 9b. Wafer-scale AiP transmitters and receivers were investigated at 60 GHz in 2016, achieving ~50% efficiency and ~15% bandwidth [126]. In 2021, Gu et al. reported the use of a wideband scalable phased array with AiP integration for 5G mmW communications [104], as shown in Figure 9c. In addition to communication, LCP-based front-end modules have been proposed for passive mmW imaging [127,128,129], as shown in Figure 9d. In 2018, researchers at the University of Delaware developed MCM front-end receivers on multilayer LCP substrates, achieving a high gain of more rate than 63 dB and a low noise rate of less than 6 dB at 95 GHz [128,129].

## 6. Other Applications

In addition to the traditional wireless applications, LCP films have been proposed in many other interesting applications, such as micro-electro-mechanical systems (MEMS) [16], biomedical devices [17], microfluidics [18], nanomaterial carriers [130,131,132], and roll-to-roll manufacturing [133]. In this section, we briefly review the first three applications, each of which has been extensively studied.

### 6.1. MEMS

**Figure 10:**
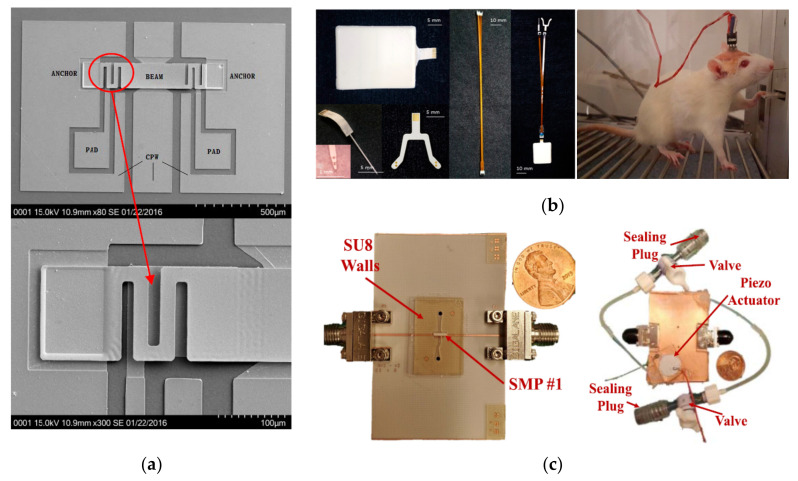
Other applications for liquid crystal polymer films. (**a**) Scanning electron microscopy images of the fabricated spring-like MEMS CPW switch from 1 to 20 GHz [137]. Copyright IEEE, 2016. (**b**) Independently fabricated biomedical modules of the neural stimulator (left) and the implantable test using a rat to characterize the stimulus parameters (right) [140]. Copyright MDPI, 2019. (**c**) Frequency-tunable bandpass filter with integrated microfluidics [141]. Copyright IEEE, 2020.

MEMS are used in microscopic devices, particularly those with moving parts, which have found attractive applications in sensing, RF switching, reconfiguration, and displays [134]. One of the most critical aspects for bringing MEMS into practical application is their packaging [16]. These small devices are typically vulnerable to contaminants and water vapors, meaning that hermetic or near-hermetic sealing is required [135]. The use of LCPs is a promising packaging approach for MEMS due to their moisture resistance, near-hermetic multilayer lamination, flexibility, and low loss tangent [16]. Among the polymeric materials, LCP has the best moisture resistance and temperature stability. Chen et al. developed a packaging enclosure for RF MEMS in 2006, which passed Method 1014, MIL-STD-883 gross leak, and fine leak hermeticity tests [135]. Jiang et al. reported the use of a laser assisted packaging method for MEMS and sensors using LCP films in 2015 [136]. The bending effects of RF MEMS, thermal actuators, and MEMS-based filters on LCP substrates were well studied by Han and colleagues [137,138,139]. A fabricated spring-like RF MEMS switch is shown in Figure 10a [137].

With the benefit of commercially available PCB technologies, MEMS-based capacitive pressure sensors were made with standard PCB fabrication [142]. In 2012, Kottapalli et al. developed an underwater MEMS pressure sensor array with near-hermetic LCP packaging [143]. Furthermore, LCP-packaged MEMS have been applied for phase shifters and AiP phased arrays [99,144].

### 6.2. Biomedical Devices

LCP films are of great importance for chronic biomedical applications due to their biocompatibility and long-term chemical resistance against most acids, bases, and solvents over a broad temperature range [17,145]. Flexible nerve stimulators with metals and iridium oxides on LCP films have been developed for retinal and cochlear implants [146,147,148,149,150,151]. In 2020, Au et al. developed an implantable optogenetic stimulator with LCP packaging, achieving a long lifetime of 326 days [150]. In 2021, Yun et al. reported the use of a remote-controlled fully implantable neural stimulator for freely moving animals with minimized power consumption, as illustrated in Figure 10b [151]. Additionally, LCP films have been applied in flexible neural probes for recording neural signals due to their relatively higher stiffness than polyimides and parylenes [140,152,153]. Lee et al. reported the use of a flexible depth-type neural probe with controllable stiffness to penetrate the dura mater of rodent brains without a guide tool or additional reinforcement structures in 2012 [152]. In 2017, a four-sided neural probe was developed to record the activities of 3-D neural networks [140]. In 2019, Jeong et al. reported on the conformal hermetic sealing of wireless microelectronic implantable chiplets [153]. A review of LCP-based neural probes is presented in [154]. On the other hand, the use of an LCP-based THz patch antenna array was reported for various medical applications, such as cancer detection and vital sign detection [155]. The long-term reliability and corresponding evaluation method of biomedical devices packaged using LCP films are discussed in [17] and [156], respectively.

### 6.3. Microfluidics

Microfluidics typically refers to the precise manipulation of fluids geometrically constrained to small scales, and has been widely applied in sensing, DNA chips, and molecular biology [157,158]. Recently, microfluidics has been developed for LCP multilayer circuits [18]. With the development of third-generation semiconductors, such as GaNs, the integration and packaging of high power MMICs have been investigated in multilayer LCP substrates with microfluidic channels as heat sinks [159,160]. Another important application of microfluidics in circuits is for reconfiguration with high linearity and high flexibility [18]. In 2014, Chlieh et al. reported the use of a tunable filter with an LCP-integrated microfluidic channel by changing the permittivity of the fluids [161]. Additionally, liquid metals moving in microfluidic channels packaged with LCP films have been proposed for antenna beam steering and frequency sweeping [162,163]. However, liquid metals have several disadvantages, such as having limited conductivity and a short lifetime [18]. To overcome these factors, movable metal plates inside microfluidic channels have been developed to control frequency-tunable antennas [164], filters [165], and array feed networks [166]. In the last two years, microfluidics integrated with piezoelectric actuator have been reported for reconfiguring single-pole, single-throw switch and hairpin filters at mmW frequencies [141,167], as shown in Figure 10c.

## 7. Outlook and Prospects

As shown in this review, the fantastic electromagnetic, mechanical, chemical, and thermal properties of LCP films are studied in many interesting research fields and interdisciplines. It is beyond our capacity to foresee all of the ideas and applications that will emerge, so we will concentrate on the newly rising fields.

Terahertz waves are sandwiched between microwaves and infrared waves in the electromagnetic spectra [168], which cover a spectral window between 0.1 and 10 THz. When compared to microwaves, terahertz waves provide high spatial resolution with a low profile, ultrahigh data transmission rate up to 100 Gbps, and free band. On the other hand, terahertz waves have a unique ability to penetrate fog, cloud, smoke cover, sand and dust storms, and thin dielectrics, which blind all current optical and IR systems. Thus, this spectral range has gained tremendous interest in various wireless applications, such as 6G communication, security scanning, remote sensing, and high-resolution radars [169]. The loss tangent of LCPs is merely 0.012 at 330 GHz [22], which is much smaller than those of FR-4 and polyimide at 10 GHz. Due to their low loss and small film thickness, LCP-based THz devices, such as SIWs [170], transition devices [171], and antennas [122,155], have been proposed for future advanced wireless systems. Furthermore, THz waves provide absorption signatures in the spectra for biomolecule vibrations, an approach that holds great promise for the detection of biomolecules and tumor cells [172]. In this regard, flexible biosensors, such as MTMs, surface plasmon polaritons [173], and antennas, fabricated on flexible LCP substrates may find important applications at THz frequencies. However, as the frequency increases, the size of the aforementioned electromagnetic devices becomes smaller so that the current fabrication resolution may face constraints. Some commercial PCB manufacturers, such as AKM Electronics Industrial (PanYu) Ltd., can provide 50 μm fabrication resolution for LCP printed circuits (even better than the traditional rigid PCBs); however, this may not be sufficient at THz frequencies as high as several hundred GHz. Liquid photoresistive techniques typically provide high fabrication resolution, which may be sacrificed due to the surface roughness of the commercial LCP films [27]. In this regard, flat LCP films with high surface uniformity and advanced PCB fabrication technologies need to be developed for THz applications.

Due to the flexibility of LCP films, electric devices on LCP films hold great promise for use in flexible electronics. LCP-based antennas, MTMs, and front-end modules have been investigated for use on curved surfaces for conformal applications, such as aircrafts, automobiles, cell phones, and human bodies [7,174], which exhibit low loss and high efficiency due to the low loss tangent of LCP films up to 1.8 THz. Moreover, the near-hermetic nature and low water absorption provide safe and bendable packages for integrated MEMS, nano-electro-mechanical systems (NEMS), MMICs, and sensors, which may be vulnerable to bad weather and corrosive chemicals. On the other hand, LCPs suitable for wearable and implantable applications due to their high chemical resistance, good biocompatibility, and low water absorption. For instance, daily disease monitoring and chronic diagnosis devices have been developed on LCP films, and neural stimulators and receivers developed on LCP substrates have been implanted in live animals for remote monitoring. Here, it is worth mentioning that LCP films may be used in THz neurology [175,176], which is an interdisciplinary field, for neural stimulation and recording in the future. Typically, the flexible substrate is more prone to bending than the conductive layer fabricated on the substrate, which means that the conductive layer mainly determines the flexibility of flexible electronic devices [3]. On the other hand, the skin depth and surface roughness of the conductors may influence the conductor loss significantly. In this regard, the thickness and material used for the conductive layer on LCP films need to be carefully designed to balance the flexibility, conductor loss, and lifetime, which may show huge differences across various frequencies and bending times.

Lastly, microfluidics technologies may be integrated in multilayer LCP films to reduce water absorption for THz sensing and to improve sensitivity for implantable biodevices. Flexible 2-D materials, such as graphene and carbon nanotubes, may be used in flexible LCP electronics for novel applications [157,177].

## Figures and Tables

**Table 1:** Global companies producing products that incorporate LCP technologies.

Classification	Company	Technologies
LCP film	muRata	BIAC Film
	kuraray	Vecstar
	SUMITOMO CHEMICAL	LCP Film (BDU)
	Superex	Oriented LCP Film
LCP Flexible Copper Clad Laminate (FCCL)	muRata	BIAC CCL
	Azotek	AZOTEX^®^-LD
	Sytech	SF701
	Rogers	ULTRALAM^®^3000/3850
	Panasonic	FELIOS-LCP, R-F705T
LCP printed circuits	muRata	MetroCire ™
	SUMITOMO ELECTRIC	High-speed circuits
	Kinwong	Flexible antennas and circuits
	MFLEX	Flexible multilayer circuits
	Fujikura	Flexible multilayer circuits
	AKM Electronics Industrial (PanYu) Ltd.	Flexible multilayer circuits
LCP modules and electronic products	muRata	mmW antenna module
	Fujikura	mmW antenna module
	Amphenol	mmW antenna module/UHD module/backplane connector
	LUXSHARE	mmW connecting line/device/antenna
	Speed	modules
	Sunway	connecting line/device/antenna
	Qualcomm	5G chip LCP RF antenna
	Apple	iPhone 7; iPhone X

## Data Availability

Not applicable.
